# Ischemic stroke with a preceding Trans ischemic attack (TIA) less than 24 hours and thrombolytic therapy

**DOI:** 10.1186/s12883-020-01782-5

**Published:** 2020-05-19

**Authors:** Nicolas Poupore, Dan Strat, Tristan Mackey, Ashley Snell, Thomas Nathaniel

**Affiliations:** University of South Carolina School of Medicine Greenville, 607 Grove Rd, Greenville, SC 29605 USA

**Keywords:** Ischemic, Stroke, Trans ischemic attack, Clinical risk factors, Thrombolytic therapy

## Abstract

**Background:**

Acute ischemic stroke attack with and without a recent TIA may differ in clinical risk factors, and this may affect treatment outcomes following thrombolytic therapy. We examined whether the odds of exclusion or inclusion for thrombolytic therapy are greater in ischemic stroke with TIA less than 24 h preceding ischemic stroke (recent-TIA) as compared to those without recent TIA or non-TIA > 24 h and less than 1 month (past-TIA).

**Methods:**

A retrospective hospital-based analysis was conducted on 6315 ischemic stroke patients, of whom 846 had proven brain diffusion-weighted magnetic resonance imaging (DW-MRI) of an antecedent TIA within 24 h prior to ischemic stroke. The logistic regression model was developed to generate odds ratios (OR) to determine clinical factors that may increase the likelihood of exclusion or inclusion for thrombolytic therapy. The validity of the model was tested using a Hosmer-Lemeshow test, while the Receiver Operating Curve (ROC) was used to test the sensitivity of our model.

**Results:**

In the recent-TIA ischemic stroke population, patients with a history of alcohol abuse (OR = 5.525, 95% CI, 1.003–30.434, *p* = 0.05), migraine (OR = 4.277, 95% CI, 1.095–16.703, *p* = 0.037), and increasing NIHSS score (OR = 1.156, 95% CI, 1.058–1.263, *p* = 0.001) were associated with the increasing odds of receiving rtPA, while older patients (OR = 0.965, 95% CI, 0.934–0.997, *P* = 0.033) were associated with the increasing odds of not receiving rtPA.

**Conclusion:**

In recent-TIA ischemic stroke patients, older patients with higher INR values are associated with increasing odds of exclusion from thrombolytic therapy. Our findings demonstrate clinical risks factors that can be targeted to improve the use and eligibility for rtPA in in recent-TIA ischemic stroke patients.

## Background

There is a general concern that Transient Ischemic Attack (TIA) patients with focal lesions, which may appear normal on computed tomography (CT), may be at higher risk for hemorrhage post-thrombolysis treatment following onset of ischemic stroke [1–5]. More than one third of symptomatic patients within the 24-h time frame may present with a number of cerebral infarcts and could be misdiagnosed and classified as a TIA [6], especially if no lesion is observed on the CT scan [7]. It has been shown that the use of CT as a diagnostic tool may not detect TIA-associated focal lesions prior to rtPA therapy in TIA-ischemic stroke patients [8]. This is because in some patients with a TIA preceding acute ischemic stroke (TIA-ischemic stroke) especially within 24 h of stroke onset, the TIA may appear invisible on CT scans, even with ischemic lesions, and this could affect treatment outcome with thrombolytic therapy [9–11].

The presence of a focal lesion on *Diffusion-weighted magnetic resonance imaging* (DW-MRI) is an important factor in the classification of TIA [12–15], and represents a contraindication for rtPA therapy [16]. The new tissue-based description of a TIA as a transient episode of neurological dysfunction without acute infarction is not a contraindication for rtPA in TIA-ischemic stroke patients [17]. We know that ischemic patients with an established TIA less than 24 h differ in clinical variables when compared with those without a TIA [2]. Patients with an established TIA more than 24 h prior to stroke will normally receive treatment and may be admitted because of the TIA, allowing a timely administration of rtPA [1, 2]. We also know that baseline clinical risk factors in recent-TIA ischemic stroke patients may affect treatment outcomes following thrombolytic therapy [18]. It is unknown whether specific clinical risk factors in ischemic stroke patients with a TIA within 24 h preceding an acute ischemic stroke may contribute to the inclusion or exclusion from rtPA therapy.

Data are lacking on the effect of specific clinical risk factors associated with inclusion or exclusion for rtPA therapy in ischemic stroke patients of a TIA that sets in within or less than 24 h preceding ischemic stroke (recent-TIA). It is possible that clinical risk factors associated with ischemic stroke patients with a TIA within or less than 24 h preceding an acute ischemic stroke may be different from ischemic stroke patients without a TIA or with an established TIA. Since a TIA within 24 h preceding ischemic stroke is not an established contraindication for rtPA, it implies that the presence or absence of specific clinical risk factors in acute ischemic stroke patients with TIA may contribute to the exclusion or inclusion of patients for rtPA therapy. In this study, we tested the hypothesis that specific baseline clinical risk factors may impact the inclusion or exclusion of recent-TIA ischemic stroke patients for rtPA therapy. The objective of the present study was to identify specific demographic and clinical risk factors associated with inclusion or exclusion from rtPA therapy in an ischemic stroke population with a TIA within 24 h preceding an acute ischemic stroke. Knowledge of such clinical risk factors would be clinically relevant in decision making, especially in developing management and treatment strategy in the population of stroke patients with established TIA within or less than 24 h preceding ischemic stroke.

## Methods

### Study population

Data from ischemic stroke patients admitted to the PRISMA Health Greenville, SC, USA between January 2010 and June 2016 were used for this study. The ethics committee of PRISMA Health approved this retrospective study. Patients that presented with ischemic stroke within 24 h of symptom onset, based on relevant ischemic lesions on CT or brain magnetic resonance image (MRI), were included in the analysis. Ischemic stroke patients were divided into two groups: the first group comprises of those with a TIA within or less than 24 h preceding ischemic stroke (recent-TIA ischemic stroke patients), while the second group consists of those with previously diagnosed TIA (at least 1 month) or no history of TIA (non-TIA < 24 h i.e. past-TIA). For the first group, identified TIA patients prior to stroke with DWI abnormalities consistent with focal lesions were excluded. In both groups, patients with symptomatic intracerebral hemorrhage (SICH) were excluded.

Data on clinical characteristics and laboratory analysis were collected directly from the stroke registry, which has been described in previous studies [19–22]. Collected data included patients’ medical history, including atrial fibrillation/atrial flutter, coronary artery disease (CAD), carotid stenosis, pregnancy, depression, diabetes, drug or alcohol abuse, dyslipidemia, family history of stroke, congestive heart failure (CHF), hormone replacement therapy (HRT), hypertension, migraine, obesity, prior stroke, prior TIA, prosthetic heart valve, peripheral vascular disease (PVD), chronic renal disease, sickle cell, sleep apnea, and history of smoking. Demographic variables data were collected include age, race and gender. Further, data on medication history and stroke severity (NIHSS) score were also obtained.

### Statistical analysis

All statistical analyses were performed using the SPSS Statistics Software version 15.0 (Chicago, IL). Descriptive statistics were used to analyze demographic and clinical risk factors in ischemic stroke patients characterized based on a TIA-24 h-ischemic stroke patients and non-TIA < 24-h patients. The Man Whitney U test, Pearson χ2 test or Student’s t test was utilized to perform a bivariate group comparison of baseline characteristics between the two groups. For continuous variables, a Student- t test was used, while Man Whitney U or Pearson’s chi squared test as appropriate was considered for analyzing discrete variables. In order to examine clinical factors associated with inclusion for rtPA, a multivariable binary logistic regression was performed.

‘Our primary exposure measures for regression analysis were clinical risk factors that are significantly associated with exclusion of inclusion for rtPA therapy. This approach provided the opportunity to determine whether the association between specific clinical risk factors in the recent-TIA ischemic stroke patients and the past-TIA patients are associated with inclusion or exclusion for rtPA. For the logistic regression, the variable selection for a multivariate model was not based on univariate *p*-values, because we considered clinical knowledge to supersede univariate statistical analysis of p-values when selecting variables for model building [23]. Therefore, the backward selection method allowed all of our variables to be selected, while removing the least significant one at each step. In general, our analysis identified variables independently associated with the increasing odds (odds ratios [OR]) and their 95% Cis of exclusion or inclusion for thrombolytic therapy in patients with and without a TIA 24 h prior to ischemic stroke.

In the regression model, rtPA treatment served as the dependent variable. In addition, variables for TIA-24 h-ischemic stroke patients were included as the primary independent for the entire ischemic stroke population with or without a recent TIA (24 h prior), established TIA (at least more than 1 month) or no TIA. The increasing odds of exclusion or inclusion in the sub-cohort for these groups were analyzed separately. A Hosmer-Lemeshow test examined the validity of the model, and the overall correct classification percentage as well as the area under the Receiver Operating Curve (ROC) for score prediction was determined. This allowed us to test the sensitivity, specificity and accuracy of our logistic model. Odds ratios (ORs) were determined from the logistic regression and significance was set at the probability level of 0.05. The odds ratio values were then utilized to predict the increasing odds of exclusion of inclusion in the TIA-24 h-ischemic stroke patients.

## Results

In this study, a total of 6315 ischemic stroke patients were identified. Of this cohort, 846 patients had a TIA within 24 h prior to the ischemic stroke, whereas 5469 did not. Table 1 presents the demographic and clinical variables of acute ischemic stroke patients with and past-TIA prior to ischemic stroke. Recent-TIA ischemic stroke patients were more likely to be white (82.2% vs. 78.4%), with lower rates of atrial fibrillation (13.9% vs 16.9%) and alcohol abuse (3.7% vs. 6.2%). The recent-TIA group also demonstrated higher rates of carotid artery stenosis (7.9% vs 6.1%) and dyslipidemia (58.2% vs 50.4%). These patients presented with higher rates of previous stroke (32.4% vs. 26.0%) and past-TIA (16.5% vs. 8.7%). Furthermore, this group was more likely to have PVD (9.9% vs. 7.3%), but less likely to smoke (20.6% vs. 27.2%). Recent-TIA ischemic stroke patients were more likely to take anti-HTN medications (73.9% vs. 69.4%), cholesterol reducers (53.9% vs. 44.4%), diabetes medications (31.8% vs. 27.3%), and antidepressants (15.5% vs. 13.0%). They presented with lower NIHSS scores (3.46 ± 4.1 vs 8.28 ± 8.24), lower total cholesterol (167.88 ± 44.01 vs 171.84 ± 51.28), lower LDL levels (98.22 ± 36.56 vs 104.62 ± 41.29), and lower blood glucose (138.59 ± 76.92 vs 147.3 ± 81.05). Finally, this group presented with a higher INR (1.19 ± 0.5 vs 1.14 ± 0.5), lower heart rate (78.02 ± 14.86 vs 82 ± 18.53), lower systolic blood pressure (148.61 ± 27.88 vs 151.82 ± 29.31), and lower diastolic blood pressure (79.37 ± 17.53 vs 82.44 ± 19.12).

**Table 1:** Demographic and clinical characteristics of ischemic stroke patients with or without a TIA < 24 h. Results for continuous variables are presented as Mean ± SD, while discrete data are presented as percentage frequency. Pearson’s Chi-Square or Man Whitney U test as appropriate is used to compare differences between demographic and clinical characteristics in groups with or without a TIA < 24 h

Demographic and clinical factors	Acute Ischemic Stroke with TIA < 24 h	Acute Ischemic Stroke without TIA < 24 h	
**Number of patients**	**846**	**5469**	**P-value**
Age Group: No. (%)			
< 50	97 (11.5)	658 (12.0)	0.299
50–59	131 (15.5)	996 (18.2)	
60–69	206 (24.3)	1299 (23.8)	
70–79	195 (23.0)	1231 (22.5)	
> =80	217 (25.7)	1285 (23.5)	
Mean ± SD	68.08 ± 14.47	67.25 ± 14.73	0.126
Race: No (%)			
White	695 (82.2)	4288 (78.4)	0.042*^a^
Black	130 (15.4)	1002 (18.3)	
Other	21 (2.5)	179 (3.3)	
Gender: No. (%)			
Female	451 (53.3)	2807 (51.3)	0.283
Male	395 (46.7)	2662 (48.7)	
Medical History: No. (%)			
Atrial Fib	118 (13.9)	924 (16.9)	0.032*^a^
Coronary Artery Disease	283 (33.5)	1661 (30.4)	0.71
Carotid Artery Stenosis	67 (7.9)	334 (6.1)	0.44*^a^
Depression	125 (14.8)	721 (13.2)	0.206
Diabetes	320 (37.8)	1935 (35.4)	0.167
Drugs or Alcohol	31 (3.7)	337 (6.2)	0.004*^a^
Dyslipidemia	492 (58.2)	2755 (50.4)	< 0.001*^a^
Stroke Family History	87 (10.3)	494 (9.0)	0.241
Heart Failure	85 (10.0)	590 (10.8)	0.516
Hormonal Replacement Therapy	11 (1.3)	79 (1.4)	0.742
Hypertension	675 (79.8)	4306 (5469)	0.485
Migraine	27 (3.2)	134 (2.5)	0.203
Obesity	356 (42.1)	2311 (42.3)	0.923
Previous Stroke	274 (32.4)	1424 (26.0)	< 0.001*^a^
Previous TIA	140 (16.5)	477 (8.7)	< 0.001*^a^
Prosthetic Heart Valve	11 (1.3)	62 (1.1)	0.673
Peripheral Vascular Disease	84 (9.9)	400 (7.3)	0.008*^a^
Chronic Renal Disease	66 (7.8)	447 (8.2)	0.713
Sickle Cell	1 (0.1)	4 (0.1)	0.665
Sleep Apnea	34 (4.0)	170 (3.1)	0.163
Smoker	174 (20.6)	1486 (27.2)	< 0.001*^a^
Medication History: No (%)			
HTN medication	625 (73.9)	3794 (69.4)	0.008*^a^
Cholesterol Reducer	456 (53.9)	2428 (44.4)	< 0.001*^a^
Diabetes medication	269 (31.8)	1495 (27.3)	0.007*a
Antidepressant	131 (15.5)	711 (13.0)	0.048*^a^
Initial NIHSS Score: No (%)			
0–9	677 (90.6)	3289 (71.7)	< 0.001*
10–14	53 (7.1)	507 (11.1)	
15–20	12 (1.6)	501 (10.9)	
21–25	5 (0.7)	290 (6.3)	
Mean ± SD	3.46 ± 4.1	8.28 ± 8.24	< 0.001*^b^
Lab values: Mean ± SD			
Total cholesterol (mg/dl)	167.88 ± 44.01	171.84 ± 51.82	0.026*^b^
Triglycerides (mg/dl)	146.38 ± 93.94	139.64 ± 105.16	0.1
HDL (mg/dl)	42.81 ± 14.28	41.78 ± 13.84	0.062
LDL (mg/dl)	98.22 ± 36.56	104.62 ± 41.29	< 0.001*^b^
Lipids (mg/dl)	7.38 ± 26.21	6.53 ± 2.56	0.389
Blood Glucose (mg/dl)	138.59 ± 76.92	147.3 ± 81.05	0.004*^b^
Serum Creatinine (mg/dl)	1.44 ± 3.45	1.29 ± 1.17	0.288
INR	1.19 ± 0.5	1.14 ± 0.5	0.036*^b^
Vital Signs: Mean ± SD			
Heart Rate	78.02 ± 14.86	82 ± 18.53	< 0.001*^b^
Blood Pressure Systolic (mmHg)	148.61 ± 27.88	151.82 ± 29.31	0.003*^b^
Blood Pressure Diastolic (mmHg)	79.37 ± 17.53	82.44 ± 19.12	< 0.001*^b^

Notes:

^a^Pearson’s Chi-Squared test

^b^Student’s T test

* *P*-value < 0.05

Table 2 presents the demographic and clinical variables of acute ischemic stroke patients with past-TIA who are divided into groups: those treated with rtPA and those without rtPA treatment. As shown in the Table 2, recent-TIA ischemic stroke patients that received rtPA were younger (57.8 ± 15.49 vs 68.39 ± 14.33), with lower rates of atrial fibrillation (0.0% vs 14.4%), but higher rates of alcohol abuse (12.0% vs. 3.41%). Recent-TIA -ischemic stroke patients that received rtPA demonstrated higher rates of migraine (16.0% vs 2.8%), obesity (68.0% vs 41.29%), higher NIHSS scores (6.16 ± 5.46 vs 3.36 ± 4.02) but were more likely to have a lower INR (0.98 ± 0.06 vs 1.19 ± 0.5). The past-TIA patients that received rtPA presented with lower rates of carotid artery stenosis (4.2% vs 6.7%), but higher rates of depression (15.4% vs. 12.5%). They demonstrated higher rates of dyslipidemia (52.8% vs 49.6%), HRT (2.3% vs 1.2%), migraine (3.4% vs 2.1%), obesity (51.1% vs 39.4%), and previous TIAs (10.8% vs. 8.1%), but were less likely to present with a previous stroke (21.9% vs 27.4%), PVD (6.0% vs 7.7%), and chronic renal disease (6.0% vs. 8.9%). The past-TIA group was more likely to take cholesterol reducers (47.6% vs. 43.4%) and antidepressants (16.7% vs. 11.8%), but less likely to take diabetes medications (24.9% vs. 28.1%). They presented with higher NIHSS scores (10.55 ± 8.18 vs 7.43 ± 8.11) but were more likely to have lower total cholesterol (168.66 ± 46.48 vs 173.01 ± 53.6). They have lower LDL levels (102.52 ± 39.07 vs 105.39 ± 42.06), lower lipids (6.25 ± 1.6 vs 6.64 ± 2.83), lower blood glucose (141.29 ± 74.84 vs 149.22 ± 82.86), and lower serum creatinine (1.14 ± 0.75 vs 1.34 ± 1.27).

**Table 2:** Demographic and clinical characteristics of ischemic stroke patients with or without a TIA < 24 h stratified by rtPA. Results for continuous variables are presented as Mean ± SD, while discrete data are presented as percentage frequency. Pearson’s Chi-Square or Man Whitney U test is used to compare differences between demographic and clinical characteristics in rtPA treated groups

Demographic and clinical factors	Acute Ischemic Stroke with TIA < 24 h			Acute Ischemic Stroke without TIA < 24 h		
	No rtPA Group	rtPA Group		No rtPA Group	rtPA Group	
Number of patients	**821**	**25**	**P-value**	**4142**	**1327**	**P-Value**
Age Group: No. (%)						
< 50 years	89 (10.84)	8 (32)	0.004*^a^	467 (11.3)	191 (14.4)	0.001*^a^
50–59	128 (15.59)	3 (12)		736 (17.8)	260 (19.6)	
60–69	197 (23.99)	9 (36)		981 (23.7)	318 (24.0)	
70–79	192 (23.38)	3 (12)		942 (22.7)	289 (21.8)	
> =80	215 (26.18)	2 (8)		1016 (24.5)	269 (20.3)	
Age Mean ± SD	68.39 ± 14.33	57.8 ± 15.49	< 0.001*^b^	67.73 ± 14.69	65.76 ± 14.78	< 0.001*^b^
Race: No (%)						
White	675 (82.21)	20 (80)	0.878	3228 (77.9)	1060 (79.9)	0.313
Black	126 (15.34)	4 (16)		774 (18.7)	228 (17.2)	
Other	20 (2.43)	1 (4)		140 (3.4)	39 (2.9)	
Gender: No. (%)						
Female	437 (53.2)	14 (56)	0.784	2148 (51.9)	659 (49.7)	0.163
Male	384 (46.77)	11 (44)		1994 (48.1)	668 (50.3)	
Medical History: No. (%)						
Atrial Fib	118 (14.4)	0 (0)	0.041*^a^	713 (17.2)	211 (15.9)	0.266
Coronary Artery Disease	275 (33.49)	8 (32)	0.876	1262 (30.5)	399 (30.1)	0.782
Carotid Artery Stenosis	65 (7.91)	2 (8)	0.988	278 (6.7)	56 (4.2)	0.001*^a^
Depression	119 (14.49)	6 (24)	0.187	516 (12.5)	205 (15.4)	0.005*^a^
Diabetes	310 (37.75)	10 (40)	0.820	1520 (36.7)	415 (31.3)	< 0.001*^a^
Drugs or Alcohol	28 (3.41)	3 (12)	0.024*^a^	260 (6.3)	77 (5.8)	0.532
Dyslipidemia	477 (58.09)	15 (60)	0.850	2055 (49.6)	700 (52.8)	0.047*^a^
Stroke Family History	82 (9.98)	5 (20)	0.104	364 (8.8)	130 (9.8)	0.265
Heart Failure	83 (10.1)	2 (8)	0.730	453 (10.9)	137 (10.3)	0.531
Hormonal Replacement Therapy	11 (1.33)	0 (0)	0.560	48 (1.2)	31 (2.3)	0.002*^a^
Hypertension	654 (79.65)	21 (84)	0.594	3262 (78.8)	1044 (78.7)	0.950
Migraine	23 (2.8)	4 (16)	0.001*^a^	89 (2.1)	45 (3.4)	0.011*^a^
Obesity	339 (41.29)	17 (68)	0.008*^a^	1633 (39.4)	678 (51.1)	< 0.001*^a^
Previous Stroke	265 (32.27)	9 (36)	0.695	1134 (27.4)	290 (21.9)	< 0.001*^a^
Previous TIA	135 (16.44)	5 (20)	0.637	334 (8.1)	143 (10.8)	0.002*^a^
Prosthetic Heart Valve	11 (1.33)	0 (0)	0.560	52 (1.3)	10 (0.8)	0.133
Peripheral Vascular Disease	83 (10.1)	1 (4)	0.314	321 (7.7)	79 (6.0)	0.029*^a^
Chronic Renal Disease	66 (8.03)	0 (0)	0.140	368 (8.9)	79 (6.0)	0.001*^a^
Sickle Cell	1 (0.12)	0 (0)	0.861	4 (0.1)	0 (0)	0.257
Sleep Apnea	32 (3.89)	2 (8)	0.304	125 (3.0)	45 (3.4)	0.495
Smoker	169 (20.58)	5 (20)	0.943	1098 (26.5)	388 (29.2)	0.052
Medication History: No (%)						
HTN medication	169 (20.58)	5 (20)	0.828	2851 (68.8)	943 (71.1)	0.125
Cholesterol Reducer	440 (53.59)	16 (64)	0.304	1796 (43.4)	632 (47.6)	0.006*^a^
Diabetes medication	260 (31.66)	9 (36)	0.647	1164 (28.1)	331 (24.9)	0.025*^a^
Antidepressant	126 (15.34)	5 (20)	0.526	489 (11.8)	222 (16.7)	< 0.001*^a^
Initial NIHSS Score: No (%)						
0–9	658 (91.13)	19 (76)	0.001*^a^	2556 (76.3)	773 (59.4)	< 0.001*^a^
10–14	50 (6.92)	3 (12)		308 (9.2)	199 (16.1)	
15–20	9 (1.24)	3 (12)		308 (9.2)	193 (15.6)	
21–25	5 (0.69)	0 (0)		180 (5.4)	110 (8.9)	
NIHSS Score Mean ± SD	3.36 ± 4.02	6.16 ± 5.46	0.018*^b^	7.43 ± 8.11	10.55 ± 8.18	< 0.001*^b^
Lab values: Mean ± SD						
Total cholesterol (mg/dl)	168.15 ± 43.98	159.45 ± 44.73	0.341	173.01 ± 53.6	168.66 ± 46.48	0.006*^b^
Triglycerides (mg/dl)	146.1 ± 94.44	154.79 ± 78.82	0.656	139.25 ± 104.13	140.71 ± 107.94	0.674
HDL (mg/dl)	42.78 ± 14.33	43.58 ± 12.75	0.787	41.77 ± 13.91	41.8 ± 13.65	0.946
LDL (mg/dl)	98.46 ± 36.6	90.79 ± 35.42	0.312	105.39 ± 42.06	102.52 ± 39.07	0.029*^b^
Lipids (mg/dl)	7.4 ± 26.61	6.55 ± 2	0.881	6.64 ± 2.83	6.25 ± 1.6	< 0.001*^b^
Blood Glucose	138.17 ± 76.44	152.16 ± 91.72	0.371	149.22 ± 82.86	141.29 ± 74.84	0.001*^b^
Serum Creatinine (mg/dl	1.45 ± 3.5	1.02 ± 0.37	0.547	1.34 ± 1.27	1.14 ± 0.75	< 0.001*^b^
INR	1.19 ± 0.5	0.98 ± 0.06	< 0.001*^b^	1.17 ± 0.57	1.06 ± 0.15	
Vital Signs: Mean ± SD						
Heart Rate	77.97 ± 14.83	79.6 ± 15.71	0.590	82.07 ± 18.94	81.81 ± 17.18	0.644
Blood Pressure Systolic (mmHg)	148.76 ± 27.95	143.6 ± 25.05	0.362	151.99 ± 30.04	151.31 ± 26.92	0.439
Blood Pressure Diastolic (mmHg)	79.24 ± 17.48	83.4 ± 18.56	0.243	82.29 ± 19.38	82.92 ± 18.3	0.277

Notes:

^a^Pearson’s Chi-Squared test

^b^Student’s T test

* *P*-value < 0.05

**Fig. 1:**
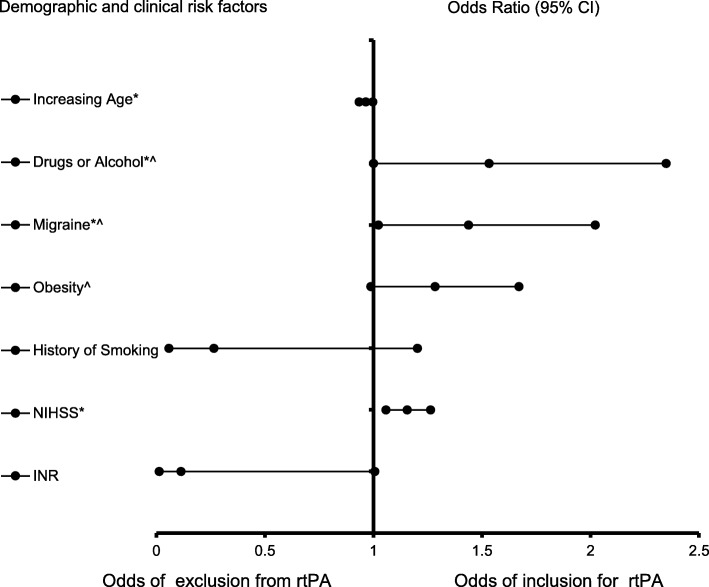
Forest Plot representation of Table 3 for factors that are associated with the odds of inclusion or exclusion for rtPA in the TIA < 24 h group. Confidence Interval band below 1 denotes factors that are associated with not receiving rtPA while Confidence Interval band above 1 denotes factors that are associated with receiving rtPA. Confidence Interval bands that cross 1 cannot be associated with either receiving or not receiving rtPA. *Indicates statistical significance (*P* < 0.05) with a 95% confidence interval. ^Indicates that data were modified by taking the 5th square root

**Fig. 2:**
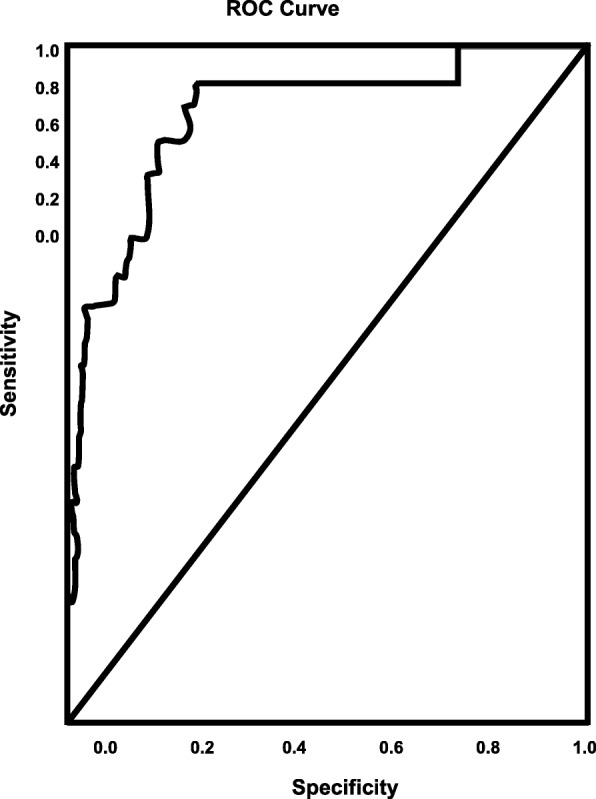
ROC curve associated with prediction of receiving rtPA for acute ischemic stroke population with TIA < 24 h. Higher area under the curve (AUC) values in ROC analysis indicates better discrimination of the score for the measured outcome. Classification table (overall correctly classified percentage = 96.1%) and area under the ROC curve (AUC = 0.895, 0.824–0.966) were applied to check model fitness

The results of logistic regression showing factors associated with rtPA in the recent-TIA ischemic stroke population is presented in Table 3, while the forest plot representation is shown in Fig. 1. The results indicate that recent-TIA ischemic stroke patients presenting with a history of alcohol abuse (OR = 5.525, 95% CI, 1.003–30.434, *p* = 0.05), migraine (OR = 4.277, 95% CI, 1.095–16.703, *p* = 0.037), and NIHSS score (OR = 1.156, 95% CI, 1.058–1.263, *p* = 0.001) were associated with increasing odds of receiving rtPA. Increasing age (OR = 0.965, 95% CI, 0.934–0.997, *P* = 0.033) and INR (OR = 0.113, 95% CI, 0.013–1.006, *p* = 0.51) were associated with increasing odds of exclusion from rtPA treatment. The ROC curve for the predictive power of the regression model is presented in Fig. 2. The discriminating capability of the model was very strong, as shown by the ROC curve, with area under the curve (AUROC) of AUROC = 0.895 (95% CI, 0.824–0.966, *P* < 0.001).

**Table 3:** Clinical factors that were associated with rtPA for ischemic stroke population with recent-TIA. Adjusted OR < 1 denote factors that are associated with not receiving rtPA while OR > 1 denote factors that are associated with receiving rtPA. Hosmer-Lemeshow test (*P* = 0.016), Cox & Snell *(R*^*2*^ = 0.679). The overall classified percentage of 96.1% was applied to check for fitness of the logistic regression model. *Indicates statistical significance (*P* < 0.05) with a 95% confidence interval

Demographic and clinical factors	B Value	Wald	Odds Ratio	95% C.I.		***P***-value
				Lower	Upper	
Increasing Age	−0.035	4.533	0.965	0.934	0.997	0.033*
Drugs or Alcohol	1.709	3.854	5.525	1.003	30.434	0.05*
Migraine	1.453	4.371	4.277	1.095	16.703	0.037*
Obesity	1.001	3.49	2.722	0.952	7.784	0.062
History of Smoking	−1.328	2.96	0.265	0.058	1.203	0.085
NIHSS	0.145	10.37	1.156	1.058	1.263	0.001*
INR	−2.181	3.82	0.113	0.013	1.006	0.051

Notes:

Backward Stepwise model based on Likelihood Ratio was applied

Model assumptions were fulfilled

Multicollinearity and interactions among independent variables were checked and no significant interactions were found

Hosmer-Lemeshow test (*P* = 0.016), Cox & Snell *(R*^*2*^ = 0.679)

**Fig. 3:**
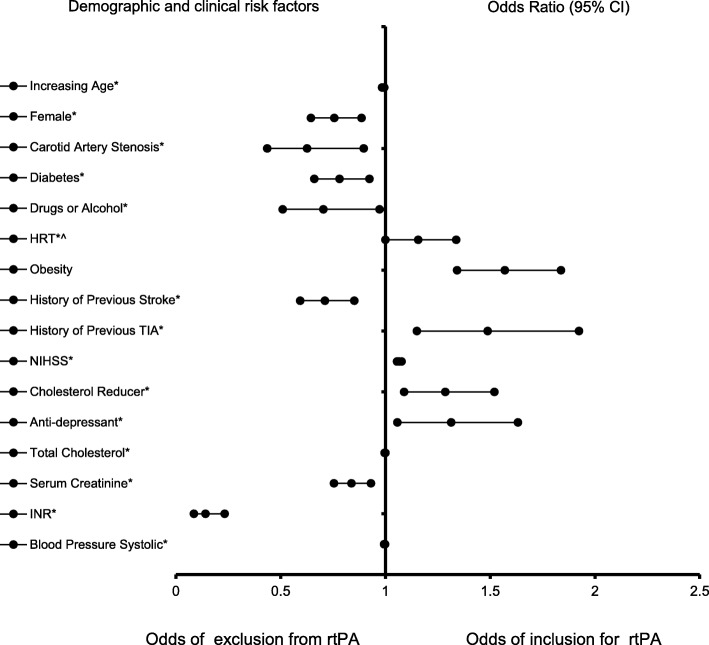
Forest Plot representation of Table 4 for factors that are associated with the odds of inclusion or exclusion for rtPA in the non-TIA < 24 h group. Confidence Interval band below 1 denotes factors that are associated with not receiving rtPA while Confidence Interval band above 1 denotes factors that are associated with receiving rtPA. Confidence Interval bands that cross 1 cannot be associated with either receiving or not receiving rtPA. *Indicates statistical significance (*P* < 0.05) with a 95% confidence interval. ^Indicates that data were modified by taking the 5th square root

**Fig. 4:**
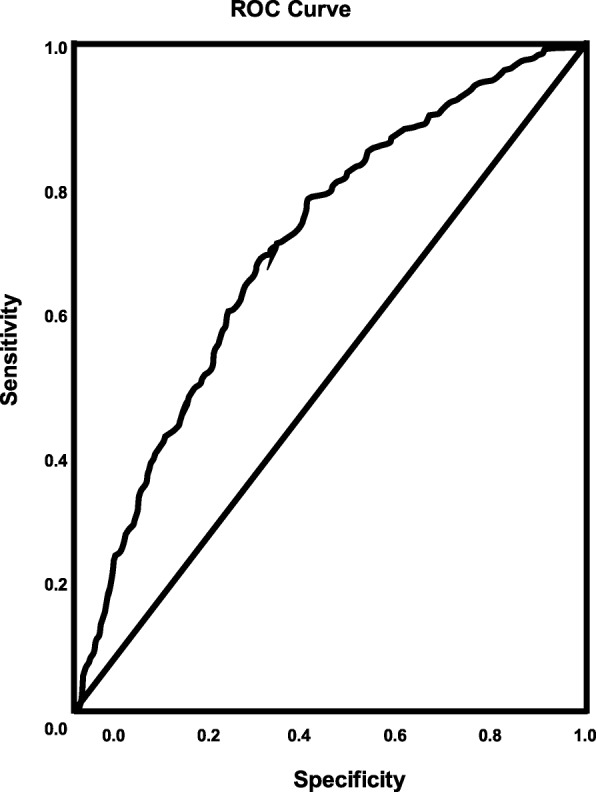
ROC curve associated with prediction of receiving rtPA for acute ischemic stroke population without a TIA < 24 h. Higher area under the curve (AUC) values in ROC analysis indicates better discrimination of the score for the measured outcome. Classification table (overall correctly classified percentage = 70.2%) and area under the ROC curve (AUC = 0.704, 0.686–0.722) were applied to check model fitness

Table 4 presents factors that are associated with the odds of receiving rtPA in the past-TIA group, while the representative forest plot is shown in Fig. 3. As shown in Table 4, HRT (OR = 1.79, 95% CI, 1.0–3.204, *p* = 0.05), obesity (OR = 1.57, 95% CI, 1.342–1.838, *p* < 0.001), history of previous TIA (OR = 1.488, 95% CI, 1.15–1.924, *p* = 0.003), NIHSS score (OR = 1.066, 95% CI, 1.056–1.077, *p* < 0.001), use of cholesterol reducers (OR = 1.286, 95% CI, 1.089–1.52, *p* = 0.003), and anti-depressant use (OR = 1.314, 95% CI, 1.057–1.633, *p* = 0.014) were associated with the increasing odds of receiving rtPA therapy. Increasing age (OR = 0.99, 95% CI, 0.984–0.995, *P* < 0.001), females (OR = 0.756, 95% CI, 0.645–0.886, *p* = 0.001), history of carotid artery stenosis (OR = 0.626, 95% CI, 0.436–0.897, *p* = 0.011), history of diabetes (OR = 0.781, 95% CI, 0.661–0.924, *p* = 0.004), alcohol abuse (OR = 0.704, 95% CI, 0.51–0.972, *p* = 0.033), history of previous stroke (OR = 0.711, 95% CI, 0.593–0.852, *p* < 0.001), total cholesterol (OR = 0.998, 95% CI, 0.996–1.0, *p* = 0.014), serum creatinine (OR = 0.838, 95% CI, 0.755–0.931, *p* = 0.001), INR (OR = 0.141, 95% CI, 0.086–0.233, *p* < 0.001), and systolic blood pressure (OR = 0.997, 95% CI, 0.994–0.999, *p* = 0.019) were associated with not receiving rtPA. As presented in Fig. 4, the predictive power of the logistic regression was strong. The area under the curve (AUROC) is 0.704 (95% CI, 0.686–0.722, *P* < 0.001).

**Table 4:** Clinical factors that were associated with receiving rtPA for ischemic stroke population past-TIA. Adjusted OR < 1 denote factors that are associated with not receiving rtPA while OR > 1 denote factors that are associated with receiving rtPA. Hosmer-Lemeshow test (*P* = 0.006), Cox & Snell *(R*^*2*^ = 0.102). The overall classified percentage of 70.2% was applied to check for fitness of the logistic regression model. *Indicates statistical significance (*P* < 0.05) with a 95% confidence interval

Demographic and clinical factors	B Value	Wald	Odds Ratio	95% C.I.		P-value
				Lower	Upper	
Increasing Age	−0.01	13.03	0.99	0.984	0.995	< 0.001*
Female	−0.28	11.977	0.756	0.645	0.886	0.001*
Carotid Artery Stenosis	−0.469	6.509	0.626	0.436	0.897	0.011*
Diabetes	−0.247	8.357	0.781	0.661	0.924	0.004*
Drugs or Alcohol	−0.351	4.548	0.704	0.51	0.972	0.033*
HRT	0.582	3.842	1.79	1	3.204	0.05*
Obesity	0.451	31.585	1.57	1.342	1.838	< 0.001*
History of Previous Stroke	−0.341	13.608	0.711	0.593	0.852	< 0.001*
History of Previous TIA	0.397	9.133	1.488	1.15	1.924	0.003*
NIHSS	0.064	168.417	1.066	1.056	1.077	< 0.001*
Cholesterol Reducer	0.252	8.759	1.286	1.089	1.52	0.003*
Anti-Depressant	0.273	6.029	1.314	1.057	1.633	0.014*
Total Cholesterol (mg/dl)	−0.002	6.06	0.998	0.996	1	0.014*
Serum Creatinine (mg/dl)	−0.176	10.9	0.838	0.755	0.931	0.001*
INR	−1.956	58.726	0.141	0.086	0.233	< 0.001*
Blood Pressure Systolic (mmHg)	−0.003	5.471	0.997	0.994	0.999	0.019*

Notes

Backward Stepwise model based on Likelihood Ratio was applied

Model assumptions were fulfilled

Multicollinearity and interactions among independent variables were checked and no significant interactions were found

Hosmer-Lemeshow test (*P* = 0.006), Cox & Snell *(R*^*2*^ = 0.102)

## Discussion

The current study investigated clinical risk factors associated with inclusion or exclusion from rtPA therapy in an ischemic stroke population presenting with a TIA within 24 h preceding an acute ischemic stroke. First, a frequency of only 13% of an antecedent TIA within 24 h in the ischemic stroke population was found. Second, a greater percentage of recent-TIA ischemic stroke patients were excluded from rtPA when compared to the past-TIA patients. Third, more clinical risk factors were associated with inclusion than exclusion for rtPA, while increasing age and INR were associated with increasing odds of exclusion from thrombolytic therapy in the recent-TIA ischemic stroke patients. Finally, more clinical risk factors were associated with exclusion than inclusion from rtPA in the ischemic stroke population in the past-TIA group.

The past-TIA ischemic stroke group analyzed in this study was older (68.08 ± 14.47) than patients reported in other studies (mean age of 63) [1]. Moreover, the recent-TIA ischemic stroke population in this study consists of a significant proportion of patients > 80 years, not excluded from rtPA in line with AHA guidelines [24] and reported to be safe by several studies [25–27]. The prevalence of a prior TIA in patients with ischemic stroke in the general population is between 7 to 40% [13, 28, 29]. The frequency of occurrence of a TIA in our recent-TIA ischemic population was 13%, which falls within the low range of occurrence among those reported [13, 28, 29]. The occurrence of a TIA within 24 h preceding the onset of acute ischemic stroke was not a contraindication for thrombolytic therapy in this study population, therefore, it is not possible that the exclusion of this group of patients is the reason for the low occurrence of TIAs observed. Since this is a retrospective study, there is the possibility of bias in data input which could potentially account for the low occurrence. However, the analysis of TIAs occurring within 24 h preceding the onset of acute ischemic stroke is mainly done by retrospective data analysis, and this method is supported by several studies as the best approach to determine an antecedent TIA [30, 31].. It is also possible that the main etiology of the ischemic stroke population with a TIA in this study is cardioembolism, which is often more severe than atherothrombotic strokes [1, 2]. Therefore, the low frequency could be attributed to lesser occurrence of stroke in a large vessel, which is more commonly indicated by temporary symptoms in contrast to stroke from cardioembolism [1].

The univariate analysis revealed that the recent-TIA ischemic stroke population that received rtPA was younger, more likely to have atrial fibrillation, have a history of alcohol abuse, be obese, present with a migraine, and have lower INR and NIH scores. Ischemic stroke patients receiving rtPA without a diagnosed TIA were younger, more likely to be depressed, have dyslipidemia, be on hormonal replacement therapy, present with a migraine, have a previous TIA, be obese, use cholesterol reducers or antidepressants, and have higher NIH scores. Following adjustment for the effect of confounding variables, hormonal replacement therapy, obesity, history of a previous TIA, and anti-depressant use were factors found to be associated with inclusion for rtPA in the past-TIA patients, while increasing age, female gender, carotid artery stenosis, diabetes, history of previous stroke and alcohol use, cholesterol, serum creatinine, INR, and increased systolic blood pressure were associated with exclusion from rtPA. The identified comorbidities associated with exclusion from thrombolytic therapy are comparable to existing prospective and retrospective studies, such as increasing age associated with worse outcomes [32], female gender corresponding with significantly higher percentages of cardioembolic strokes [33], and severe carotid artery stenosis, which predicts poor outcome [34]. Other identified comorbidities reported in previous studies are diabetes; due to hyperglycemia, which increases the risk of cerebral hemorrhage with rtPA treatment [35], and chronic alcohol use; which increases excitotoxic/ischemic damage, leading to poor outcomes [36]. History of previous stroke represents an exclusion criteria, especially in patients with large infarctions and concern of bleeding [37]. Furthermore, high levels of serum creatinine as an exclusion criteria is due to risk of symptomatic intracranial hemorrhage [38], while elevated INR is due to the *risk* of intracranial hemorrhage [39]. Finally, higher peak values of systolic blood pressure may lead to higher rates of hemorrhagic complications [40].

A transient ischemic attack preceding ischemic stroke does not appear to have a major influence on outcomes following rtPA, and may actually induce neuroprotection in patients with ischemic stroke [41]. This argues for the necessity to understand the other clinical risk factors which might affect thrombolytic treatment outcome. This study revealed that TIA-24 h-ischemic stroke patients with a history of alcohol abuse are more likely to be included for rtPA. Alcohol consumption as a risk factor for stroke is known to follow a J-shaped curve, such that modest drinkers (< 15 g/d) have the lowest risk, and heavy drinkers (> 60 g/d) have the highest [42]. The signs and symptoms of acute alcohol intoxication are similar to those of vertebrobasilar stroke [43]. Due to similarities in symptoms such as double vision, nystagmus, dysarthria, and ataxia, the differential diagnosis of alcohol intoxication versus vertebrobasilar stroke may constitute a major diagnostic challenge. Moreover, if alcohol intoxication and stroke occur concurrently, the signs and symptoms of stroke may be linked to the effects of alcohol, resulting in a delayed stroke diagnosis and failure to administer thrombolytic therapy. In the case of uncertainty, and if stroke cannot be excluded, thrombolytic therapy can be administered [43]. It is possible that the TIA-24 h-ischemic stroke patients with a history of alcohol abuse arrived at the hospital early, allowing stroke diagnosis to be established within the therapeutic time window. Therefore, the recent-TIA ischemic stroke patients with a simultaneous history of alcohol abuse were more likely to be eligible for rtPA therapy.

Furthermore, the recent-TIA ischemic stroke patients that presented with migraine were more likely to receive rtPA therapy. Migraine has been established as the third most common stroke mimic and contributes to greater than 17% of poor outcomes in thrombolytic therapy [44]. Thrombolytic therapy in ischemic stroke patients with migraine is associated with a low risk of poor treatment outcome [44]. With an increased capability of accurately diagnosing a migraine attack, it is possible that a comprehensive clinical evaluation provided an accurate diagnosis of migraine with existing history in the TIA-ischemic stroke patients in this study. Even when a definitive diagnosis is not possible, the evidence of rtPA safety in scientific literature [45–49] supports its administration, as shown in the current study.

Another major finding in this study is that older recent-TIA ischemic stroke patients are more likely to be excluded from rtPA. A TIA is known to have negligible effects on patients 50 years below, but significantly reduces life expectancy in individuals 65 years and older [50]. Compared to patients 50 years and younger, patients that are 75–84 years old are at a greater risk. Furthermore, patients above the age of 80 have the highest risk [50]. In line with this finding, the study showed that elderly stroke patients with a TIA within 24 h prior to stroke may be excluded from rtPA therapy, suggesting that these patients may have the most to gain from intensive cerebrovascular risk management.

A major challenge with a TIA is the uncertainty of its diagnosis, including the neurovascular implication, which is systemic in many TIA studies [51]. More than 25% of patients assumed to present with a TIA in the emergency department were further assessed and noted to have had a stroke, while 25% had an established mimic condition [52]. While we could not dependably differentiate among stroke cases misclassified as a TIA, or complications of stroke mimicking a TIA in our retrospective data, our sample best represents those progressing to stroke after a TIA presentation assessed by DW-MRI prior to onset of ischemic stroke. Therefore, our retrospective data consist of ischemic stroke patients diagnosed with a TIA within 24 h prior to onset of ischemic stroke, reflecting real world clinical scenarios. It has been shown that an estimated 70% of patients with a diagnosis of a TIA are admitted to the hospital and, of these, only 50% retain a TIA diagnosis [52, 53]. The studied cohort represents those retaining a TIA diagnosis within 24 h prior to stroke, reducing misclassification. A major limitation of this study is that the results cannot be generalized to ischemic stroke patients with a TIA diagnosis greater than 24 h prior to stroke. In addition, the small number of ischemic stroke patients having a TIA within 24 h prior to onset of stroke could be considered another limitation of the study in the demonstration of differences in clinical risk factors between ischemic stroke with a TIA < 24 h prior and ischemic stroke without a TIA < 24 h prior. Moreover, there is no data for onset time to arrival.

However, in the light of 13% occurrence of a TIA < 24 h prior to ischemic stroke in this population, which fits within the existing range of between 7 to 40% [1, 2, 28], it seems likely that these findings will be clinically relevant in decision making with respect to the management of clinical risk factors associated with thrombolytic therapy.

## Conclusion

Patients presenting with a TIA less than 24 h prior to an ischemic stroke were more likely to be excluded from receiving rtPA than those without a TIA 24 h prior, with the most likely cause of exclusion criteria being increased age. Considering the small number of patients presenting with a TIA within 24 h prior to an ischemic stroke and the high exclusion rate from rtPA within this group, this study provides vital information that can help facilitate future clinical decisions when administering rtPA to TIA-24 h-ischemic stroke patients.

## Data Availability

The retrospective datasets are available by request from the corresponding author of this manuscript.

## Abbreviations

rtPA: Recombinant tissue plasminogen activator
TIA: Trans ischemic attack
OR: Adjusted odd ratio
GWTG: Get With The Guideline
AHA: American Heart Association
NIH scores: National Institute of Health scores
AUROC: Area under the curve
ROC: Receiver operator characteristic
CI: Confidence interval
CAD: Coronary artery disease
MAP: Mean arterial pressure
CHF: Congestive heart failure
PVD: Peripheral vascular disease
DW-MRI: Diffusion-weighted magnetic resonance imaging
HRT: Hormone replacement therapy
CT: Computed tomography
MRI: Magnetic resonance image
